# Transmission of Zika virus through breast milk and other breastfeeding-related bodily-fluids: A systematic review

**DOI:** 10.1371/journal.pntd.0005528

**Published:** 2017-04-10

**Authors:** Susannah Colt, Maria N. Garcia-Casal, Juan Pablo Peña-Rosas, Julia L. Finkelstein, Pura Rayco-Solon, Zita C. Weise Prinzo, Saurabh Mehta

**Affiliations:** 1 Division of Nutritional Sciences, Cornell University, Ithaca, New York, United States of America; 2 Evidence and Programme Guidance, Department of Nutrition for Health and Development, World Health Organization, Geneva, Switzerland; University of Heidelberg, GERMANY

## Abstract

**Background:**

Zika virus (ZIKV) infection is an emerging mosquito-borne disease, which is associated with an increase in central nervous system malformations and newborn microcephaly cases. This review investigated evidence of breastfeeding transmission from ZIKV-infected mothers to their children and the presence of ZIKV infection in breastfeeding-related fluids.

**Methodology/Principal findings:**

We conducted a systematic review of observational studies, case studies, and surveillance reports involving breastfeeding women with ZIKV infection in several international databases. Data extraction and analysis were conducted following a PROSPERO-registered protocol. From 472 non-duplicate records, two case reports met criteria for inclusion. We reviewed three cases of ZIKV infection among lactating mothers near the time of delivery. Two of the three (2/3) associated newborns had evidence of ZIKV infection. ZIKV was detected in breast milk of all three mothers. Breast milk detection results were positive in all mothers (3/3) by RT-PCR, one was positive by culture (1/3), and none was tested for ZIKV-specific antibodies. Serum samples were ZIKV positive in all mothers (3/3), and sweat was not tested for ZIKV.

**Conclusions/Significance:**

We describe three cases of ZIKV-infected breastfeeding mothers who were symptomatic within three days of delivery, and two cases with ZIKV-infected newborns. While ZIKV was detected in the breast milk of all three mothers, the data are not sufficient to conclude ZIKV transmission via breastfeeding. More evidence is needed to distinguish breastfeeding transmission from other perinatal transmission routes.

## Author summary

Zika virus (ZIKV) infection was considered a mild fever illness until the recent and ongoing outbreak in South America revealed that the virus can cause microcephaly and other neurological disorders. ZIKV is primarily transmitted by mosquitoes, but can also spread from person to person through sex, blood transfusion, and from mother to child during pregnancy or delivery. It is unknown if ZIKV can spread from mother to child during breastfeeding. We conducted a systematic review of the literature to summarize evidence of ZIKV transmission via breastfeeding and the presence ZIKV in breast milk. Our search resulted in 2 case reports that described 3 mothers and their newborns. ZIKV was confirmed in the blood and breast milk of all 3 mothers and in the blood of 2 newborns. More evidence is needed to confirm if ZIKV is transmitted via breastfeeding.

## Introduction

Zika virus (ZIKV) infection is an emerging vector-borne disease of the *Flaviviridae* family, which includes dengue, yellow fever, Japanese encephalitis, and West Nile viruses [1]. ZIKV infection causes a mild, self-limiting influenza-like illness with a 10-day incubation for most cases and shares similarities with other circulating arthropod-borne viral infections like the alphavirus chikungunya [1, 2]. Many cases of ZIKV infection are asymptomatic and therefore unreported.

**Fig 1:**
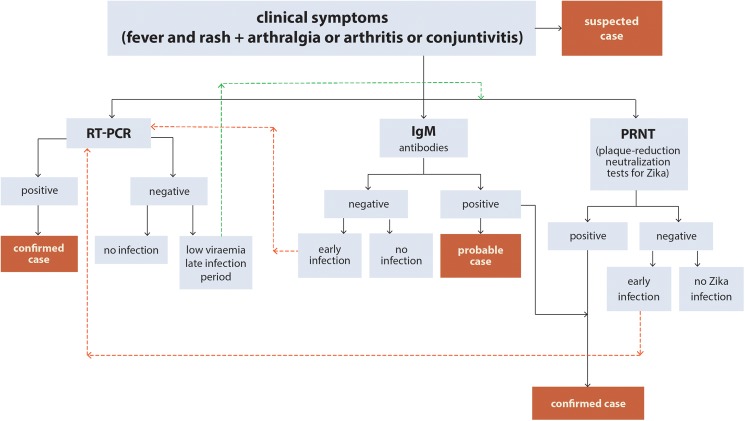
Case definitions and main diagnostic tests interpretations for Zika virus.

The World Health Organization (WHO) has developed an interim case definition to classify and report cases of ZIKV infection (Fig 1). A **suspect case** is a person presenting with rash and/or fever and at least one of the following: arthralgia, arthritis or conjunctivitis. A **probable case** is a suspected case with presence of IgM antibody against ZIKV and an epidemiological link; and a **confirmed case** is a person with laboratory confirmation of recent ZIKV infection: by presence of ZIKV RNA or antigen in serum or other samples **or** IgM antibody against ZIKV positive and plaque reduction neutralization test ≥ 90% (PRNT_90_) for ZIKV with titre ≥ 20 and ZIKV PRNT_90_ titre ratio ≥ 4 compared to other flaviviruses [3]. Due to the possible cross reactivity with other members of the *Flaviviridae* family, the presence of IgM is not enough to rule out ZIKV infection, and the PRNT_90_ will determine if the *in vitro* inhibition of cell growth is produced by antibodies against ZIKV [4, 5]. An enzyme linked immunoassay (ELISA) for ZIKV has been developed by the Centers for Disease Control and Prevention, but is only available upon request for emergency use [6].

The timing and the test performed could be crucial for detecting the ZIKV infection. During the first 7 days, viral RNA can often be identified by reverse transcriptase polymerase chain reaction (RT-PCR), but as viremia decreases, a negative RT-PCR does not exclude flavivirus infection, and serologic testing should be performed. On the other hand virus-specific IgM antibodies may be detectable >4 days after onset of illness, however a sample taken within 7 days of illness onset may not have detectable virus-specific IgM antibodies [7].

ZIKV transmission occurs primarily via the bite of *Aedes aegypti* mosquitoes, in addition to *Aedes* spp. *Ae*. *africanus*, *Ae*. *albopictus*, *Ae*. *hensilli*, and *Ae*. *Luteocephalus* [2, 8–12]. However, perinatal, transfusion, and sexual transmission have also been reported [13–17]. Among infected individuals, evidence of ZIKV has been detected in serum, saliva, urine, semen, and breast milk [13, 18–22]. Generally, transmission of antibodies through breast milk has been described, particularly for IgA, conferring passive immunity [23]. The presence of IgA, IgG, or IgM antibodies against similar flaviviruses such as West Nile Virus has been reported in breast milk [24]. Given recent increases of ZIKV cases in Central and South America and suggested associations with congenital microcephaly and other non-congenital neurological or autoimmune disorders, an investigation of transmission via breast milk is needed [25].

Until recently, outbreaks of ZIKV were sporadic. During the last 50 years, widespread infection throughout Africa and Southeast Asia is suspected, but the asymptomatic nature and limited diagnostics have likely hampered disease surveillance [12, 26–28]. In 2007, the disease migrated to Oceania where an outbreak in Yap State in the Federated States of Micronesia infected roughly 5,000 individuals, nearly 75% of the island population [2]. The next outbreaks occurred in French Polynesia (396 confirmed), New Caledonia (1,400 confirmed), and the Cook Islands (50 confirmed) in 2013–2014 [29–31]. The first official outbreak in the Americas arrived to Easter Island, Chile in early 2014 with 51 confirmed cases [32]. In April, 2015, Brazil reported the first confirmed autochthonous case of ZIKV infection [33]. Since then, an epidemic has rapidly expanded affecting 48 countries and territories in South and Central America [34]. The Brazilian Ministry of Health estimates the number of ZIKV cases in 2015 alone between 0.4–1.3 million [8].

During the Brazilian ZIKV epidemic, clinicians have observed a 20-fold increase in suspected cases of microcephaly in newborns [35]. Reported microcephaly and/or central nervous system malformations have affected 7,150 individuals in Brazil between 22 October 2015 and 16 April 2016 [36]. Other flaviviruses have not been known to cause microcephaly, however ZIKV has been confirmed in recent microcephaly cases, which has prompted global concern for pregnant women and a large-scale investigation [37]. A recent report from the WHO indicated that there is scientific consensus that Zika virus is a cause of microcephaly and Guillain-Barré syndrome [36, 38] based on results from a systematic review [39, 40]. Many mothers of infants with microcephaly reported no illness or symptoms associated with Zika infection [41]. Regardless of symptoms, pregnant women are at risk for infection and potential complication in any trimester [13, 42]. At this time, the WHO recommends standard breastfeeding practices for all mothers, regardless of ZIKV infection [43], unless there is an acceptable medical reason for permanent or temporary avoidance of breastfeeding [44].

The primary objective of this systematic review was to review evidence related to the transmission of ZIKV through breastfeeding. For the purposes of this review, ZIKV infection included suspected, probable, or confirmed cases as described by the WHO interim case definition. A secondary objective assessed the available literature regarding the presence of ZIKV or ZIKV-specific antibodies in breast milk and breastfeeding-related bodily fluids (i.e. blood or sweat) of lactating women. We sought to address the following questions:

Primary Outcome: Does the literature provide evidence that in ZIKV-free infants or children, breastfeeding (any or exclusive) from a ZIKV-infected lactating mother, compared to not breastfeeding, result in evidence of ZIKV infection in the infant?

Secondary Outcome: Does the literature provide evidence there are ZIKV specific antibodies present in breast milk?

## Methods

### Study criteria

Study characteristics, as well as inclusion and exclusion criteria, were defined by study designs, participants, ZIKV infection exposure, and outcomes.

#### Types of studies

We included observational studies, case studies, and surveillance reports, which include epidemiological data from outbreak investigations.

#### Types of participants

Study participants were limited to adolescents age 10–19 years or adult women who were lactating, or expressing milk, with ZIKV infection. This includes lactating participants who were currently breastfeeding or not, as well as those who were breastfeeding prior to a ZIKV presumptive diagnosis. Studies with populations who did not meet these criteria or who had a non-ZIKV infection were excluded.

#### Types of exposure

Exposure criteria were described as any mothers with ZIKV infection who were breastfeeding or expressing breast milk.

#### Types of outcomes

Primary outcomes included infants or children with any ZIKV infection (suspected, probable or confirmed cases), within 30 days of breastfeeding or receiving expressed breast milk from a mother with ZIKV infection. Secondary outcomes included the detection of ZIKV in breast milk, maternal blood, maternal sweat or infant saliva by the detection methods that allow identification as suspected, probable and confirmed cases. Detection methods included:

- ZIKV RNA by reverse transcriptase polymerase chain reaction (RT-PCR)
- ZIKV-specific IgM antibody by ELISA
- PRNT_90_ for ZIKV with titre > 20 and ZIKV PRNT_90_ titre ratio > 4 compared to other flaviviruses
- ZIKV isolation in culture

### Search strategy

A search overview is provided in the S1 Appendix.

Electronic databases: Search terms included variations and permutations of United States National Library of Medicine Medical Subject Headings (MeSH) terms and text words relating to flaviviruses (Zika, West Nile, and yellow fever), breastfeeding, transmission fluids (breast milk, blood, and sweat), and participants (mother or child) (See appendix for full search strategy). Report characteristics included a time range of all years, any language, and any publication status. The following electronic databases were searched:

- MEDLINE & MEDLINE in Process (OVID) 1946 to 9 March 2016
- PubMed
- CINAHL (Ebsco) 1982 to March 2016
- Web of Science (ISI) SCI, SSCI, CPCI & CPCI-SSH to 2 March 2016
- Popline to March 2016
- LILACS (Birme) 1982 to March 2016
- PAHO (Birme) to March 2016
- WHOLIS (Birme) to March 2016
- WPRIM to March 2016
- IMSEAR to March 2016

#### Additional search strategy

To identify ongoing and unpublished studies or case reports, we searched the WHO International Clinical Trials Registry Platform (ICTRP - http://apps.who.int/trialsearch/Default.aspx), and the PAHO Zika Research Portal (http://www.paho.org/zika-research/index.php) separately (11 March 2016). We also contacted CDC and the WHO and PAHO Zika outbreak teams for recent or unpublished findings (11 March 2016). The references cited in the included studies were also reviewed for potential selection of studies. We contacted authors of the identified studies for additional information on their published reports.

### Study selection

Screening of search results was performed using Covidence systematic review software (Veritas Health Innovation, Melbourne, Australia). Two authors independently screened the titles and abstracts of studies based on the inclusion criteria. A third author assessed and resolved disagreements on study selection. All irrelevant titles were excluded. For studies that met eligibility criteria, full text articles were obtained and managed using EndNote (version X7·5 2016 Thomson Reuters), a reference management software.

### Data extraction and management

A data extraction form was tailored for this review. One author extracted study characteristics and two authors extracted study outcome data according to the pre-designed data extraction form. For each study, information pertaining to the source, eligibility, methods, participants, exposures, outcomes, and results was entered into the data extraction form. When relevant, effect estimates including odds ratios, relative risks, mean differences, or summary effects were extracted for each outcome. All potential modifiers or confounders of study outcomes were included in the extraction form.

This review followed a pre-established protocol based on methods for systematic reviews described in the Cochrane Handbook for Systematic Reviews [45]. The protocol was registered in PROSPERO, the international prospective register of systematic reviews of the University of York and the National Institute for Health Research, under the number CRD42016036667. The authors followed Preferred Reporting Items for Systematic Reviews and Meta-Analyses (PRISMA) guidelines and include a checklist in the S1 Checklist.

#### Quality of the evidence

We set out the main findings of the review in Summary of Findings tables prepared using GRADE profiler software (GRADEpro Guideline Development Tool, McMaster University, 2015, developed by Evidence Prime, Inc.). The primary outcomes were listed with estimates of relative effects along with the number of participants and studies contributing data for the outcomes. For each individual outcome, we assessed the quality of the evidence using the GRADE handbook for grading quality of evidence [46]. We expressed the results as one of four levels of quality (high, moderate, low, or very low).

## Results

**Fig 2:**
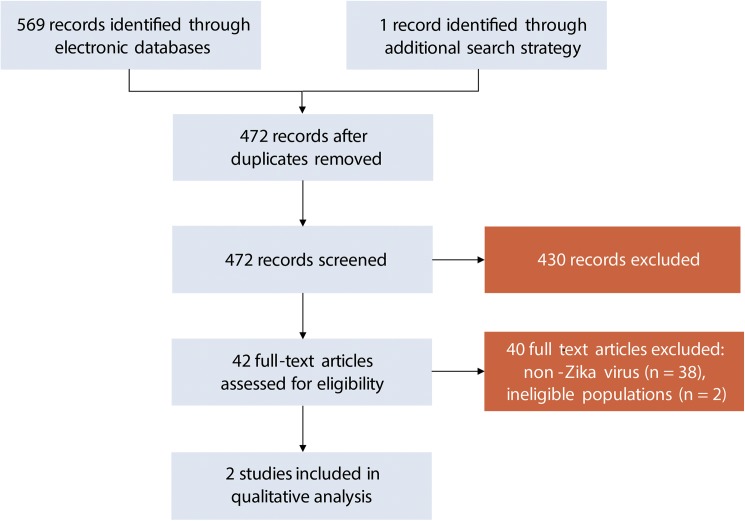
Systematic review process.

Our search strategy (11 March 2016) identified 472 records, detailed in the S1 Table, after duplicates were removed (Fig 2). No unpublished records were identified in the search nor included in the analysis. Initial screening retained 42 records. At the time of screening, inclusion criteria included terms for West Nile and yellow fever viruses. For the purposes of this review, only ZIKV was considered for quantitative analyses, which yielded 2 records for review (Fig 2). A total of 2 studies (that included three mother child pairs) were included for analysis. The main reasons for exclusion were non-ZIKV infections or ineligible populations.

A case report from the ZIKV outbreak in French Polynesia (French territory) described two mothers, who had recently given birth, with ZIKV infection (Table 1) [13]. Mother 1 initiated breastfeeding to Newborn 1 on the day of delivery. On day 2 following delivery, mother 1 had a confirmed case of ZIKV detected by serum RT-PCR and saliva RT-PCR. On day 3, the breast milk from mother 1 was found to contain ZIKV by RT-PCR, however ZIKV breast milk culture was negative. Also on day 3, Newborn 1 had confirmed ZIKV infection by serum RT-PCR and saliva RT-PCR.

**Table 1:** Detection of ZIKV in breastfeeding mother-infant pairs.

Case	Days After Delivery	Maternal					Newborn		
		Clinical	Serum	Saliva	Breast Milk		Clinical	Serum	Saliva
		Sign	RT-PCR^1^	RT-PCR	RT-PCR	Culture	Sign	RT-PCR	RT-PCR
1	-2	Rash	-	-	-	-	-	-	-
	0	-	-	-	-	-	-	-	-
	1	Rash	-	-	-	-	-	-	-
	2	Rash	Positive	Positive	-	-	-	-	-
	3	-	-	-	Positive	Negative	-	Positive	Positive
2	0	-	-	-	-	-	-	Negative	-
	1	-	Positive	-	-	-	-	-	-
	3	Rash, Mild fever	-	-	-	-	-	Negative	-
	4	-	-	-	-	-	Rash	Positive	-
	5	-	Positive	-	-	-	-	-	-
	7	-		-	-	-	-	Positive	-
	8	-	Negative	-	Positive	Negative	-	-	-
	11	-	Negative	-	-	-	-	-	-
	13	-	Negative	-	-	-	-	-	-
3	0	Fever	-	-	-	-	-	-	-
	1	Fever	-	-	-	-	-	-	-
	3	-	Positive	-	-	-	-	Ambiguous	-
	4	-	-	-	Positive	Positive	-	-	-

^1^ RT-PCR = Reverse transcription polymerase chain reaction

Mother 2 was confirmed with ZIKV infection on days 1 and 5 post delivery by serum RT-PCR and initiated breastfeeding on day 3. On day 8, the ZIKV RT-PCR results from mother 2 were serum negative, urine positive, and breast milk positive, however ZIKV breast milk culture was negative. Newborn 2 tested negative for ZIKV on day 0 and day 3 by serum RT-PCR, but had confirmed ZIKV infection on days 4 and 7 by serum RT-PCR and on day 8 by urine RT-PCR. On day 9, newborn 2 urine was ZIKV negative by RT-PCR. These case reports confirmed ZIKV infection in 2 breastfeeding mothers and their newborns as well as detected ZIKV in serum and breast milk of both mothers. Both mothers had clinical signs of rash within days of delivery, and the authors hypothesized that the infants were probably infected in utero or intrapartum because the infants’ sera were positive for the presence of Zika virus within one day of starting breastfeeding. The author of this study was contacted (M. Besnard, personal communication, 2016) and confirmed that no long-term complications were reported for either of the two infants at 2 years of age.

A second case report described a mother (referred to as case 3 in Table 1) from New Caledonia (French territory) who initiated breastfeeding on the day of delivery and developed fever and maculopapular rash in the following days [47]. On day 3 post delivery, mother 3 tested positive for ZIKV infection by serum RT-PCR, however the serum RT-PCR results for newborn 3 were reported as ambiguous. Breast milk was ZIKV positive by RT-PCR on day 4 and ZIKV breast milk culture was also positive. While vertical transmission was not described in this case, the presence of ZIKV in breast milk was confirmed. No long-term complications were reported for the child at 8 months of age (M. Dupont-Rouzeyrol, personal communication, 2016). The overall quality of the evidence was very low for all the proposed outcomes, as described in the GRADE Summary of Findings (Table 2).

**Table 2:** GRADE Summary of Findings.

Breastfeeding (any or exclusive) from a lactating woman with suspected, probable or confirmed Zika virus infection compared to not breastfeeding in infants and young children						
Patient or population: infants and young children						
Setting: areas of Zika virus transmission						
Intervention: breastfeeding from mothers with suspected, probable or confirmed Zika virus infection						
Comparison: not breastfeeding from mothers with suspected, probable or confirmed Zika virus infection						
Outcomes	Anticipated absolute effects* (95% CI)		Relative effect (95% CI)	Number of participants (studies)	Quality of the evidence (GRADE)	Comments
	Risk with not breastfeeding from mothers infected with Zika Virus	Risk with breastfeeding from mothers infected with Zika virus				
Suspected Zika virus infection among infants or young children breastfeeding from mothers with Zika virus infection	—	1/3 (33.3%)		3 (2 observational studies)	⨁◯◯◯ VERY LOW^1^	Based on the WHO interim case definition to classify and report cases of Zika virus infection [38], a suspect case is a person presenting with rash and/or fever and at least one of the following: arthralgia, arthritis or conjunctivitis. Given the difficulty of determining arthralgia, arthritis or conjunctivitis among infants, we considered presentation with fever or rash among infants born to mothers with suspected, probably or confirmed Zika virus infection as a suspect case. Mother 1 initiated breastfeeding to Newborn 1 on the day of delivery. She was a confirmed case of Zika virus infection detected by serum RT-PCR and saliva RT-PCR on day 2. Newborn 1 did not develop symptoms though had confirmed Zika virus infection by serum RT-PCR and saliva RT-PCR on day 3 [13]. Mother 2 was confirmed with Zika virus infection on days 1 and 5 postdelivery by serum RT-PCR and initiated breastfeeding on day 3. Newborn 2 had a rash on day 4 and was subsequently confirmed to have Zika virus infection on day 4 by serum RT-PCR and on day 8 by urine RT-PCR [13]. Newborn 2 was considered a suspect case (prior to confirmation by RT-PCR). Mother 3 initiated breastfeeding to Newborn 3 on the day of delivery. She was a confirmed case of Zika virus infection detected by serum RT-PCR on day 3. Newborn 3 did not develop fever or rash [47].
Probable Zika virus infection among infants or young children breastfeeding from mothers with Zika virus infection	—	—				A **probable case** is a suspected case with presence of immunoglobulin M (IgM) antibody against the Zika virus and an epidemiological link. None of the three newborn infants were tested for IgM against the Zika virus by enzyme-linked immunosorbent assay [13, 47].
Confirmed Zika virus infection among infants or young children breastfeeding from mothers with Zika virus infection	—	2/3 (66.7%)	—	3 (2 observational studies)	⨁◯◯◯ VERY LOW^1^	Mother 1 initiated breastfeeding to Newborn 1 on the day of delivery. On day 2 following delivery, Mother 1 had a confirmed case of Zika virus infection detected by serum RT-PCR and saliva RT-PCR. On day 3, Newborn 1 had confirmed Zika virus infection by serum RT-PCR and saliva RT-PCR [13]. Mother 2 was confirmed with Zika virus infection on days 1 and 5 postdelivery by serum RT-PCR and initiated breastfeeding on day 3. Newborn 2 tested negative for Zika virus on day 0 and day 3 by serum RT-PCR, but had confirmed Zika virus infection on days 4 and 7 by serum RT-PCR and on day 8 by urine RT-PCR [13]. Both mothers had clinical signs of rash within days of delivery, and the authors concluded that vertical Zika virus transmission probably occurred during vaginal delivery [13]. Mother 3 initiated breastfeeding on the day of delivery and developed fever and maculopapular rash in the following days. On day 3 postdelivery, Mother 3 tested positive for Zika virus infection by serum RT-PCR and test results for Newborn 3 were reported as ambiguous [47]. The data are not sufficient to conclude that the transmission of the virus from the two mothers to the two infected infants was through breastfeeding. Other considerations include transmission through perinatal routes (in utero or during delivery).
Presence of Zika virus in breast milk (RT-PCR) of mothers who are acutely ill with confirmed Zika virus infection	—	3/3 (100.0%)	—	3 (2 observational studies)	⨁◯◯◯ VERY LOW^1^	Mother 1 had a confirmed case of Zika infection detected by serum RT-PCR and saliva RT-PCR on day 2 after delivery. On day 3, the breast milk from Mother 1 was found, by RT-PCR, to contain Zika virus [13]. Mother 2 was confirmed with Zika infection on days 1 and 5 post-delivery by serum RT-PCR On day 8, the Zika virus RT-PCR results from Mother 2 were positive in the breast milk [13]. Mother 3 tested positive for Zika infection by RT-PCD on day 3 postdelivery. Breast milk from Mother 3 was positive for Zika virus by RT-PCR on day 4 [47]. Because of the documented presence of Zika virus RNA (detected through RT-PCR) in breast milk, breast milk may be considered as potentially infectious. However, there are currently no documented reports of Zika virus being transmitted to infants through breast milk or breastfeeding.
Culture of Zika virus from breast milk of mothers who are acutely ill with confirmed Zika virus infection	—	1/3 (33.3%)	—	3 (2 observational studies)	⨁◯◯◯ VERY LOW^1^	Cultures of breast milk from Mothers 1 and 2 were negative for Zika virus [13]. Breast milk culture was positive for Zika virus from the breast milk of Mother 3 on day 4 after delivery [47]. Because of the documented presence of replicative Zika virus (detected in cell culture) in breast milk, breast milk may be considered as potentially infectious. However, there are currently no documented reports of Zika virus being transmitted to infants through breast milk or breastfeeding.

CI: Confidence interval; RT-PCR: reverse transcription polymerase chain reaction

*The risk in the intervention group (and its 95% CI) is based on the assumed risk in the comparison group and the relative effect of the intervention (and its 95% CI).

GRADE Working Group grades of evidence

High quality: We are very confident that the true effect lies close to that of the estimate of the effect

Moderate quality: We are moderately confident in the effect estimate: The true effect is likely to be close to the estimate of the effect, but there is a possibility that it is substantially different

Low quality: Our confidence in the effect estimate is limited: The true effect may be substantially different from the estimate of the effectVery low quality: We have very little confidence in the effect estimate: The true effect is likely to be substantially different from the estimate of effect

^1^ The evidence is based on three mother–infant pairs from two case-reports. A case report of two mother-infant pairs was from the Zika virus outbreak in French Polynesia from 2013 to 2014 (Mother 1 and Mother 2) [13]. The second case report was from the Zika virus outbreak in New Caledonia in 2015 (Mother 3) [47].

## Discussion

The cases presented in these two reports confirm the presence of ZIKV RNA in breast milk from three ZIKV-infected mothers. The presence of Zika-specific antibodies was not reported in these cases. Of the three newborns delivered to ZIKV-infected mothers who were receiving breast milk with confirmed presence of ZIKV, only two were confirmed to be infected with ZIKV with no reported adverse outcomes. With regard to the presence of ZIKV in breastfeeding-related fluids, ZIKV was detected by RT-PCR in breast milk and blood of the three mothers; sweat was not measured.

Like other viral infections, mother-to-child transmission of ZIKV infection can potentially occur during antepartum, intrapartum, or postnatal periods.[48] Given the variable incubation period for ZIKV, it can be difficult to distinguish breastfeeding transmission from other perinatal routes. For the two newborns who contracted ZIKV from ZIKV-infected mothers expressing ZIKV-infected breast milk, antepartum or intrapartum transmission is suspected. Even if a newborn is ZIKV negative following delivery from a ZIKV-infected mother and contracts ZIKV infection while consuming breast milk with ZIKV, there remains a possibility for separate mosquito transmission. Identifying the time of infection and duration of an incubation period is further complicated by the asymptomatic nature of acute ZIKV infection.

There is limited evidence describing breastfeeding transmission for other flavivirus infections. West Nile virus (WNV), dengue virus, and yellow fever virus have been detected in breast milk [49, 50]. Of these infections, WNV has been associated with breastfeeding transmission in a small number of cases [24]. Like ZIKV, breastfeeding transmission for other flavivirus infections is likely underreported due to asymptomatic illness and limited access to diagnostics. We intend to review breastfeeding transmission for related flavivirus infections in the near future.

Our systematic review for ZIKV breastfeeding transmission resulted in two studies and three cases of lactating women with confirmed ZIKV infection. As new data emerges from these current outbreaks, further investigation is needed to explore ZIKV breastfeeding transmission dynamics. This includes understanding the mechanics of transmission with regards to timing of infection for mother and infant, breast milk viral load, and exposure duration as well as assessing the frequency and distribution of breastfeeding transmission among affected populations. In addition to determining viral transmission risk, research should also explore the protective properties of ZIKV-specific immunoglobulin in breast milk transferred from mothers who have experienced ZIKV infection. At this time, the data are not sufficient to conclude ZIKV transmission via breastfeeding, and the authors support the WHO breastfeeding guidelines currently in place recommending initiating breastfeeding within one hour of delivery, exclusively for 6 months and extended until 2 years or beyond [51].

## Supporting information

S1 Checklist PRISMA Checklist. (DOC)

S1 Appendix Search Overview. (DOCX)

S1 Table Search Results. (DOCX)

## References

[1] Hayes EB. Zika virus outside Africa. Emerging infectious diseases. 2009;15(9):1347–50. Epub 2009/10/01. PubMed Central PMCID: PMCPMC2819875. doi: 10.3201/eid1509.090442 19788800 10.3201/eid1509.090442 PMC2819875

[2] Duffy MR, Chen TH, Hancock WT, Powers AM, Kool JL, Lanciotti RS, et al Zika virus outbreak on Yap Island, Federated States of Micronesia. The New England journal of medicine. 2009;360(24):2536–43. doi: 10.1056/NEJMoa0805715 19516034 10.1056/NEJMoa0805715

[3] World Health Organization. Zika virus disease: Interim case definition. 12 February 2016. Report No.: WHO/ZIKV/SUR/16.1.

[4] Timiryasova TM, Bonaparte MI, Luo P, Zedar R, Hu BT, Hildreth SW. Optimization and validation of a plaque reduction neutralization test for the detection of neutralizing antibodies to four serotypes of dengue virus used in support of dengue vaccine development. The American journal of tropical medicine and hygiene. 2013;88(5):962–70. PubMed Central PMCID: PMCPMC3752766. doi: 10.4269/ajtmh.12-0461 23458954 10.4269/ajtmh.12-0461 PMC3752766

[5] World Health Organization Department of Immunization VaB. Guidelines for plaque reduction neutralization testing of human antibodies to dengue viruses. Geneva: World Health Organization, 2007 Contract No.: WHO/IVB/07.07.

[6] New CDC laboratory test for zika virua authorized for emergency use by FDA [Internet]. Centers for Disease Control and Prevention,; 2016. Media Statement 26 February 2016

[7] Revised diagnostic testing for Zika, chikungunya, and dengue viruses in US Public Health Laboratories [Internet]. 2016. Memorandum 7 February 2016

[8] Zika virus epidemic in the Americas: potential association with microcephaly and Guillain-Barré syndrome. 10 12 2015 [Internet]. Stockholm: European Centre for Disease Prevention and Control,; 2015. Rapid Risk Assessment

[9] Haddow AJ, Williams MC, Woodall JP, Simpson DI, Goma LK. Twelve Isolations of Zika Virus from Aedes (Stegomyia) Africanus (Theobald) Taken in and above a Uganda Forest. Bulletin of the World Health Organization. 1964;31:57–69. Epub 1964/01/01. PubMed Central PMCID: PMCPMC2555143. 14230895 PMC2555143

[10] Ledermann JP, Guillaumot L, Yug L, Saweyog SC, Tided M, Machieng P, et al Aedes hensilli as a potential vector of Chikungunya and Zika viruses. PLoS neglected tropical diseases. 2014;8(10):e3188 Epub 2014/10/10. PubMed Central PMCID: PMCPMC4191940. doi: 10.1371/journal.pntd.0003188 25299181 10.1371/journal.pntd.0003188 PMC4191940

[11] Lee VH, Moore DL. Vectors of the 1969 yellow fever epidemic on the Jos Plateau, Nigeria. Bulletin of the World Health Organization. 1972;46(5):669–73. Epub 1972/01/01. PubMed Central PMCID: PMCPMC2480796. 4403105 PMC2480796

[12] Marchette NJ, Garcia R, Rudnick A. Isolation of Zika virus from Aedes aegypti mosquitoes in Malaysia. The American journal of tropical medicine and hygiene. 1969;18(3):411–5. Epub 1969/05/01. 4976739 10.4269/ajtmh.1969.18.411

[13] Besnard M, Lastere S, Teissier A, Cao-Lormeau V, Musso D. Evidence of perinatal transmission of Zika virus, French Polynesia, December 2013 and February 2014. Euro surveillance: bulletin Europeen sur les maladies transmissibles = European communicable disease bulletin. 2014;19(13). 24721538

[14] Foy BD, Kobylinski KC, Chilson Foy JL, Blitvich BJ, Travassos da Rosa A, Haddow AD, et al Probable non-vector-borne transmission of Zika virus, Colorado, USA. Emerging infectious diseases. 2011;17(5):880–2. Epub 2011/05/03. PubMed Central PMCID: PMCPMC3321795. doi: 10.3201/eid1705.101939 21529401 10.3201/eid1705.101939 PMC3321795

[15] Musso D, Cao-Lormeau VM, Gubler DJ. Zika virus: following the path of dengue and chikungunya? Lancet (London, England). 2015;386(9990):243–4. Epub 2015/07/22. 10.1016/S0140-6736(15)61273-9 26194519

[16] Musso D, Nhan T, Robin E, Roche C, Bierlaire D, Zisou K, et al Potential for Zika virus transmission through blood transfusion demonstrated during an outbreak in French Polynesia, November 2013 to February 2014. Euro surveillance: bulletin Europeen sur les maladies transmissibles = European communicable disease bulletin. 2014;19(14). Epub 2014/04/18. 10.2807/1560-7917.es2014.19.14.20761 24739982

[17] Epidemiological Update: Neurological syndrome, congenital anomalies, and Zika virus infection. 17 1 2016 [Internet]. Washington D.C.: Pan American Health Organization and World Health Organization,; 2016

[18] Lanciotti RS, Kosoy OL, Laven JJ, Velez JO, Lambert AJ, Johnson AJ, et al Genetic and serologic properties of Zika virus associated with an epidemic, Yap State, Micronesia, 2007. Emerging infectious diseases. 2008;14(8):1232–9. Epub 2008/08/06. PubMed Central PMCID: PMCPMC2600394. doi: 10.3201/eid1408.080287 18680646 10.3201/eid1408.080287 PMC2600394

[19] Musso D, Roche C, Nhan TX, Robin E, Teissier A, Cao-Lormeau VM. Detection of Zika virus in saliva. Journal of clinical virology: the official publication of the Pan American Society for Clinical Virology. 2015;68:53–5. 26071336 10.1016/j.jcv.2015.04.021

[20] Barzon L, Pacenti M, Berto A, Sinigaglia A, Franchin E, Lavezzo E, et al Isolation of infectious Zika virus from saliva and prolonged viral RNA shedding in a traveller returning from the Dominican Republic to Italy, January 2016. Euro surveillance: bulletin Europeen sur les maladies transmissibles = European communicable disease bulletin. 2016;21(10):30159. 26987769 10.2807/1560-7917.ES.2016.21.10.30159

[21] Gourinat AC, O'Connor O, Calvez E, Goarant C, Dupont-Rouzeyrol M. Detection of Zika virus in urine. Emerging infectious diseases. 2015;21(1):84–6. PubMed Central PMCID: PMCPMC4285245. doi: 10.3201/eid2101.140894 25530324 10.3201/eid2101.140894 PMC4285245

[22] Musso D, Roche C, Robin E, Nhan T, Teissier A, Cao-Lormeau VM. Potential sexual transmission of Zika virus. Emerging infectious diseases. 2015;21(2):359–61. Epub 2015/01/28. PubMed Central PMCID: PMCPMC4313657. doi: 10.3201/eid2102.141363 25625872 10.3201/eid2102.141363 PMC4313657

[23] Bridgman SL, Konya T, Azad MB, Sears MR, Becker AB, Turvey SE, et al Infant gut immunity: a preliminary study of IgA associations with breastfeeding. J Dev Orig Health Dis. 2016;7(1):68–72. doi: 10.1017/S2040174415007862 26690933 10.1017/S2040174415007862

[24] Hinckley AF, O'Leary DR, Hayes EB. Transmission of West Nile virus through human breast milk seems to be rare. Pediatrics. 2007;119(3):e666–71. doi: 10.1542/peds.2006-2107 17332186 10.1542/peds.2006-2107

[25] Zika virus infection outbreak, Brazil and the Pacific region. 25 5 2015 [Internet]. Stockholm: European Centre for Disease Prevention and Control,; 2015. Rapid Risk Assessment

[26] Fagbami AH. Zika virus infections in Nigeria: virological and seroepidemiological investigations in Oyo State. The Journal of hygiene. 1979;83(2):213–9. Epub 1979/10/01. PubMed Central PMCID: PMCPMC2129900. 489960 10.1017/s0022172400025997 PMC2129900

[27] Moore DL, Causey OR, Carey DE, Reddy S, Cooke AR, Akinkugbe FM, et al Arthropod-borne viral infections of man in Nigeria, 1964–1970. Annals of tropical medicine and parasitology. 1975;69(1):49–64. Epub 1975/03/01. 1124969 10.1080/00034983.1975.11686983

[28] Simpson DI. Zika Virus Infection in Man. Transactions of the Royal Society of Tropical Medicine and Hygiene. 1964;58:335–8. Epub 1964/07/01. 14175744

[29] Cao-Lormeau VM, Roche C, Teissier A, Robin E, Berry AL, Mallet HP, et al Zika virus, French polynesia, South pacific, 2013. Emerging infectious diseases. 2014;20(6):1085–6. Epub 2014/05/27. PubMed Central PMCID: PMCPMC4036769. doi: 10.3201/eid2006.140138 24856001 10.3201/eid2006.140138 PMC4036769

[30] Dupont-Rouzeyrol M, O'Connor O, Calvez E, Daures M, John M, Grangeon JP, et al Co-infection with Zika and dengue viruses in 2 patients, New Caledonia, 2014. Emerging infectious diseases. 2015;21(2):381–2. Epub 2015/01/28. PubMed Central PMCID: PMCPMC4313662. doi: 10.3201/eid2102.141553 25625687 10.3201/eid2102.141553 PMC4313662

[31] Roth A, Mercier A, Lepers C, Hoy D, Duituturaga S, Benyon E, et al Concurrent outbreaks of dengue, chikungunya and Zika virus infections—an unprecedented epidemic wave of mosquito-borne viruses in the Pacific 2012–2014. Euro surveillance: bulletin Europeen sur les maladies transmissibles = European communicable disease bulletin. 2014;19(41). Epub 2014/10/28. 10.2807/1560-7917.es2014.19.41.20929 25345518

[32] Tognarelli J, Ulloa S, Villagra E, Lagos J, Aguayo C, Fasce R, et al A report on the outbreak of Zika virus on Easter Island, South Pacific, 2014. Archives of virology. 2016;161(3):665–8. Epub 2015/11/28. doi: 10.1007/s00705-015-2695-5 26611910 10.1007/s00705-015-2695-5

[33] World Health Organization. Zika virus infection–Brazil and Colombia. Disease Outbreak News [Internet]. 2015. Available from: http://www.who.int/csr/don/21-october-2015-zika/en/.

[34] World Health Organization. Zika virus microcephaly and Guillain-Barré Syndrome. Situation Report: 17 November 2016 [Internet]. Available from: http://apps.who.int/iris/bitstream/10665/251462/1/zikasitrep17Nov16-eng.pdf?ua=1.

[35] Gulland A. WHO urges countries in dengue belt to look out for Zika. BMJ (Clinical research ed). 2016;352:i595. Epub 2016/01/31. 10.1136/bmj.i595 26825803

[36] World Health Organization. Zika virus microcephaly and Guillain-Barré Syndrome. Situation Report: 21 April 2016 [Internet]. Available from: http://apps.who.int/iris/bitstream/10665/205505/1/zikasitrep_21Apr2016_eng.pdf?ua=1.

[37] Recognizing, Managing, and Reporting Zika Virus Infections in Travelers Returning from Central America, South America, the Caribbean, and Mexico. 15 January 2016 [Internet]. Centers for Disease Control and Prevention,; 2016. Available from: http://emergency.cdc.gov/han/han00385.asp

[38] World Health Organization. Zika causality statement [updated 7 September 2016]. Available from: http://www.who.int/emergencies/zika-virus/causality/en/.

[39] Cao-Lormeau VM, Blake A, Mons S, Lastere S, Roche C, Vanhomwegen J, et al Guillain-Barre Syndrome outbreak associated with Zika virus infection in French Polynesia: a case-control study. Lancet (London, England). 2016;387(10027):1531–9. 10.1016/S0140-6736(16)00562-6 PMC5444521 26948433

[40] Rasmussen SA, Jamieson DJ, Honein MA, Petersen LR. Zika Virus and Birth Defects—Reviewing the Evidence for Causality. The New England journal of medicine. 2016;374(20):1981–7. doi: 10.1056/NEJMsr1604338 27074377 10.1056/NEJMsr1604338

[41] Dyer O. Zika virus spreads across Americas as concerns mount over birth defects. BMJ (Clinical research ed). 2015;351:h6983. Epub 2015/12/25. 10.1136/bmj.h6983 26698165

[42] Oliveira Melo AS, Malinger G, Ximenes R, Szejnfeld PO, Alves Sampaio S, Bispo de Filippis AM. Zika virus intrauterine infection causes fetal brain abnormality and microcephaly: tip of the iceberg? Ultrasound in obstetrics & gynecology: the official journal of the International Society of Ultrasound in Obstetrics and Gynecology. 2016;47(1):6–7. Epub 2016/01/06. 10.1002/uog.15831 26731034

[43] World Health Organization. Breastfeeding in the context of Zika virus. 25 February 2016. Report No.: Contract No.: WHO/ZIKV/MOC/16.5.

[44] World Health Organization. Acceptable medical reasons for use of breast-milk substitutes. 2009 WHO/NMH/NHD/09.01. 24809113

[45] Higgins JPT, Green S. Cochrane Handbook for Systematic Reviews of Interventions: The Cochrane Collaboration; 2011 Version 5.1.0, updated March 2011.

[46] GRADE handbook for grading quality of evidence and strength of recommendations. Schünemann H, Brożek J, Guyatt G, Oxman A, editors: The GRADE Working Group; 2013.

[47] Dupont-Rouzeyrol M, Biron A, O'Connor O, Huguon E, Descloux E. Infectious Zika viral particles in breastmilk. Lancet (London, England). 2016;387(10023):1051. 10.1016/S0140-6736(16)00624-3 26944028

[48] Kesho Bora Study G. Maternal HIV-1 disease progression 18–24 months postdelivery according to antiretroviral prophylaxis regimen (triple-antiretroviral prophylaxis during pregnancy and breastfeeding vs zidovudine/single-dose nevirapine prophylaxis): The Kesho Bora randomized controlled trial. Clinical infectious diseases: an official publication of the Infectious Diseases Society of America. 2012;55(3):449–60. PubMed Central PMCID: PMCPMC3393708. 22573845 10.1093/cid/cis461 PMC3393708

[49] Centers for Disease Control and Prevention. Transmission of yellow fever vaccine virus through breast-feeding—Brazil, 2009. MMWR Morbidity and mortality weekly report. 2010;59(5):130–2. Epub 2010/02/13. 20150888

[50] Barthel A, Gourinat AC, Cazorla C, Joubert C, Dupont-Rouzeyrol M, Descloux E. Breast milk as a possible route of vertical transmission of dengue virus? Clinical infectious diseases: an official publication of the Infectious Diseases Society of America. 2013;57(3):415–7. Epub 2013/04/12. 23575200 10.1093/cid/cit227

[51] World Health Organization. Infant and young child feeding. Fact sheet N°342 [updated January 2016, Accessed on April 19, 2016]. Available from: http://www.who.int/mediacentre/factsheets/fs342/en/.

