# Supplementary material for: Transmission of Zika virus through breast milk and other breastfeeding-related bodily-fluids: A systematic review
Source: PLoS Negl Trop Dis. 2017 Apr 10;11(4):e0005528. doi: 10.1371/journal.pntd.0005528 (PMC5398716; doi:10.1371/journal.pntd.0005528)
Supplement: S1 Appendix — (DOCX) [file pntd.0005528.s002.docx]

**S1 Appendix. Search Overview**

MEDLINE and Medline in Progress (OVID)

1 Flavivirus/ (1367)

2 Flavivirus Infections/ (506)

3 zika.tw. (333)

4 (Flaviviridae or Flavivirus).tw. (4045)

5 ZIKV.tw. (53)

6 West Nile Fever/ (3381)

7 West Nile virus/ (3775)

8 Yellow Fever/ (2564)

9 Yellow fever virus/ (1148)

10 (yellow fever* or west nile* or Kunjin).tw. (10075)

11 exp Breast Feeding/ (31065)

12 Milk, Human/ (16293)

13 (((breastfeed* or breast) adj2 feed*) or lactat*).tw. (138436)

14 ((breast or human or mother*) adj2 milk).mp. [mp=title, abstract, original title, name of substance word, subject heading word, keyword heading word, protocol supplementary concept word, rare disease supplementary concept word, unique identifier] (24003)

15 or/11-14 (172163)

16 or/1-10 (14313)

17 Sweat/ (3545)

18 exp Blood/ (990614)

19 Mucus/ (8604)

20 Saliva/ (34692)

21 (sweat* or blood* or mucus or serum* or sera or fluid* or saliva).tw. (2582998)

22 Infectious Disease Transmission, Vertical/ (12798)

23 Antibodies, Viral/ (66607)

24 or/17-23 (3289755)

25 mothers/ (31502)

26 exp Infant/ (996971)

27 (mother* or infant* or baby or babies or newborn*).tw. (557321)

28 or/25-27 (1234144)

29 24 and 28 (198442)

30 15 and 16 (55)

31 16 and 29 (267)

32 30 or 31 (308)

CINAHL (EBSCO)

S30 S28 OR S29

S29 S13 AND S27

S28 S13 AND S14

S27 S22 AND S26

S26 S23 OR S24 OR S25

S25 (mother* or infant* or baby or babies or newborn*)

S24 (MH "Infant+")

S23 (MH "Mothers")

S22 S15 OR S16 OR S17 OR S18 OR S19 OR S20 OR S21

S21 (MH "Antibodies, Viral")

S20 (MH "Disease Transmission, Vertical")

S19 (sweat* or blood* or mucus or serum* or sera or fluid* or saliva)

S18 (MH "Saliva")

S17 (MH "Mucus")

S16 (MH "Blood+")

S15 (MH "Sweat")

S14 S9 OR S10 OR S11 OR S12

S13 S1 OR S2 OR S3 OR S4 OR S5 OR S6 OR S7 OR S8

S12 ((breast or human or mother*) N2 milk)

S11 (((breastfeed* or breast) N2 feed*) or lactat*)

S10 (MH "Milk, Human")

S9 (MH "Breast Feeding+")

S8 (yellow fever* or west nile* or Kunjin)

S7 (MH "Yellow Fever")

S6 (MH "West Nile Virus")

S5 (MH "West Nile Fever")

S4 ZIKV

S3 Flaviviridae or Flavivirus

S2 zika

S1 (MH "Flavivirus") OR (MH "Flavivirus Infections+")

Web of Science (SCI, SSCI, CPCI & CRCI-SSH) (ISI)

# 14 #13 OR #12

Indexes=SCI-EXPANDED, SSCI, CPCI-S, CPCI-SSH Timespan=All years

# 13 #11 AND #4

Indexes=SCI-EXPANDED, SSCI, CPCI-S, CPCI-SSH Timespan=All years

# 12 #8 AND #4

Indexes=SCI-EXPANDED, SSCI, CPCI-S, CPCI-SSH Timespan=All years

# 11 #10 AND #9

Indexes=SCI-EXPANDED, SSCI, CPCI-S, CPCI-SSH Timespan=All years

# 10 TOPIC: (((mother* or infant* or baby or babies or newborn*)))

Indexes=SCI-EXPANDED, SSCI, CPCI-S, CPCI-SSH Timespan=All years

# 9 TOPIC: (((sweat* or blood* or mucus or serum* or sera or fluid* or saliva)))

Indexes=SCI-EXPANDED, SSCI, CPCI-S, CPCI-SSH Timespan=All years

# 8 #7 OR #6 OR #5

Indexes=SCI-EXPANDED, SSCI, CPCI-S, CPCI-SSH Timespan=All years

# 7 TOPIC: ((((breast or human or mother*) near/2 milk)))

Indexes=SCI-EXPANDED, SSCI, CPCI-S, CPCI-SSH Timespan=All years

# 6 TOPIC: ((lacat*))

Indexes=SCI-EXPANDED, SSCI, CPCI-S, CPCI-SSH Timespan=All years

# 5 TOPIC: ((((breastfeed* or breast) near/2 (feed*))))

Indexes=SCI-EXPANDED, SSCI, CPCI-S, CPCI-SSH Timespan=All years

# 4 #3 OR #2 OR #1

Indexes=SCI-EXPANDED, SSCI, CPCI-S, CPCI-SSH Timespan=All years

# 3 TOPIC: ((("yellow fever*" or "west nile*" or Kunjin)))

Indexes=SCI-EXPANDED, SSCI, CPCI-S, CPCI-SSH Timespan=All years

# 2 TOPIC: ((Flaviviridae or Flavivirus))

Indexes=SCI-EXPANDED, SSCI, CPCI-S, CPCI-SSH Timespan=All years

# 1 TOPIC: ((zika))

Indexes=SCI-EXPANDED, SSCI, CPCI-S, CPCI-SSH Timespan=All years

Popline

(Zika OR Flaviviridae OR Flavivirus OR yellow fever OR west nile OR Kunjin) AND (mother OR women)

PAHO, WHOLIS and LILACS (BIRME)

zika or yellow fever$ or west nile$ or Kunjin or Flaviviridae or Flavivirus [Words] and sweat$ or blood$ or mucus or serum$ or sera or fluid$ or saliva or breastfeed$ or breast feed$ or lactat$ or breast milk$ or mother$ milk or human milk [Words] and mother$ or infant$ or baby or babies or newborn$ [Words]

IMSEAR & WPRIM

zika or yellow fever or west nile or Kunjin or Flaviviridae or Flavivirus
