# Supplementary material for: Transmission of Zika virus through breast milk and other breastfeeding-related bodily-fluids: A systematic review
Source: PLoS Negl Trop Dis. 2017 Apr 10;11(4):e0005528. doi: 10.1371/journal.pntd.0005528 (PMC5398716; doi:10.1371/journal.pntd.0005528)
Supplement: S1 Table — (DOCX) [file pntd.0005528.s003.docx]

**S1 Table. Search Results**

| **Database searched** | **Date searched** | **Number of results** |
| --- | --- | --- |
| MEDLINE & MEDLINE in Process (OVID) 1946 to 9/3/16 | 10-Mar-2016 | 308 |
| CINAHL (Ebsco) 1982 to March 2016 | 10-Mar-2016 | 82 |
| Web of Science (ISI) SCI, SSCI, CPCI & CPCI-SSH to 2/3/16 | 10-Mar-2016 | 94 |
| Popline to March 2016 | 10-Mar-2016 | 20 |
| LILACS (Birme) 1982 to March 2016 | 10-Mar-2016 | 14 |
| PAHO (Birme) to March 2016 | 10-Mar-2016 | 0 |
| WHOLIS (Birme) to March 2016 | 10-Mar-2016 | 0 |
| WPRIM to March 2016 | 10-Mar-2016 | 0 |
| IMSEAR to March 2016 | 10-Mar-2016 | 2 |
| **Total** | | **569** |
| **After de-duplication** | | **471** |
